# A recombinase polymerase amplification assay for rapid detection of Crimean-Congo Haemorrhagic fever Virus infection

**DOI:** 10.1371/journal.pntd.0006013

**Published:** 2017-10-13

**Authors:** Laura C. Bonney, Robert J. Watson, Babak Afrough, Manija Mullojonova, Viktoriya Dzhuraeva, Farida Tishkova, Roger Hewson

**Affiliations:** 1 National Infection Service, Public Health England, Porton Down, Salisbury, United Kingdom; 2 Department of Virology, Tajik Research Institute of Preventive Medicine of the Ministry of Health of the Republic of Tajikistan, Dushanbe, Republic of Tajikistan; University of Texas Medical Branch, UNITED STATES

## Abstract

**Background:**

Crimean-Congo Haemorrhagic fever Virus (CCHFV) is a rapidly emerging vector-borne pathogen and the cause of a virulent haemorrhagic fever affecting large parts of Europe, Africa, the Middle East and Asia.

**Methodology/principle findings:**

An isothermal recombinase polymerase amplification (RPA) assay was successfully developed for molecular detection of CCHFV. The assay showed rapid (under 10 minutes) detection of viral extracts/synthetic virus RNA of all 7 S-segment clades of CCHFV, with high target specificity. The assay was shown to tolerate the presence of inhibitors in crude preparations of mock field samples, indicating that this assay may be suitable for use in the field with minimal sample preparation. The CCHFV RPA was successfully used to screen and detect CCHFV positives from a panel of clinical samples from Tajikistan.

**Conclusions/significance:**

The assay is a rapid, isothermal, simple-to-perform molecular diagnostic, which can be performed on a light, portable real-time detection device. It is ideally placed therefore for use as a field-diagnostic or in-low resource laboratories, for monitoring of CCHF outbreaks at the point-of-need, such as in remote rural regions in affected countries.

## Author summary

The Crimean-Congo Haemorrhagic fever (CCHF) recombinase polymerase amplification assay is a new, rapid and portable diagnostic method. It has been developed for the detection of infection with CCHF virus (CCHFV), the cause of a deadly haemorrhagic disease in humans and an emerging global health threat. As a rapid diagnostic suitable for use on a portable and lightweight detection device, this has the potential to be used for fast-turnaround diagnosis at the point of need, providing timely results to clinicians at the bedside and preventing the spread of virus in the hospital setting. As a portable diagnostic it would also enable the diagnosis of CCHFV to be taken out of the laboratory during an outbreak, enabling testing in field laboratories or community clinics.

## Introduction

CCHFV is an RNA virus classified in the *Orthonairovirus* genus, of the family *Nairoviridae*, within the order *Bunyavirales (*International Committee on Taxonomy of Viruses 2016). It displays a high degree of sequence variability [1] with the S-segments of CCHF viruses being phylogenetically grouped into 7 clades (Europe 1, Europe 2, Africa 1, Africa 2, Africa 3, Asia 1 and Asia 2) [2][3][4].

CCHF is easily transmitted by close contact and causes a virulent haemorrhagic fever in humans for which there is no effective prophylaxis or treatment: in consequence it is classified by the Advisory Committee on Dangerous Pathogens (ACDP) as a Hazard Group 4 pathogen mandating maximum microbiological containment (containment level 4; CL4). CCHFV has been detected in large parts of the globe including Southern and Eastern Europe, Central Asia, Western China, the Middle East and most of Africa [1] [5] [6]. CCHF has been described as “one of the most rapidly emerging viral haemorrhagic fevers in Africa, Asia and Eastern Europe,” by the WHO, with case numbers increasing in many countries in recent years [7] [8] [9]. The case fatality for CCHF is typically between 5–50%, but has been documented as high as 73% [1] [10] [11]. Ticks of the genus *Hyalomma* function as vector as well as natural reservoir of CCHFV [12]. The *Hyalomma* tick feeds on various vertebrate hosts and as a consequence CCHFV is carried by wild animals and livestock [13] [14] and can be transmitted to humans both by tick bite and by contact with infected bodily fluids [1].

With outbreaks occurring in rural areas; commonly in low-resource regions with limited access to conventional laboratory facilities, there is an urgent need for a simple, fast, reliable and portable diagnostic test [8]. This would enable rapid diagnosis and public health management of suspect human cases, as well as surveillance of the virus in vertebrate and tick populations in isolated locations. CCHFV infection has a propensity for nosocomial transmission, especially during the early stages of disease when symptoms are poorly recognised and laboratory diagnosis is not commonly requested or performed. In these circumstances it can lead to outbreaks of highly pathogenic disease sustained by human-to-human transmission [8] [10] [12]. Rapid detection of positive cases could lead to more timely and appropriate support for patients, including their isolation to prevent transmission and the protection of health care providers by initiation of barrier nursing techniques. More convenient tools for CCHFV surveillance in the environment would also facilitate our understanding of the natural fluxes of virus in populations and help develop effective countermeasures and timely interventions [8].

There has been a recent proliferation of research into next-generation molecular diagnostics with improvements in performance relative to traditional PCR [15] [16] [17] [18] [19] [20] [21] [22] [23] [24] [25] [26]. Many of these adopt a single (isothermal) incubation temperature and a variety of non-thermal methods to separate duplex DNA, allowing amplification of a target region without the need for thermal cycling. Recombinase polymerase amplification (RPA) is a well-established isothermal molecular technique and compares favourably with other isothermal methods such as LAMP (Loop-mediated isothermal amplification), with a rapid turnaround time and simple set-up. It is performed at a single low temperature (37–42 degrees), using a recombinase enzyme to separate the DNA duplex and single-stranded DNA-binding proteins to stabilise the open complex [16], allowing amplification and detection with standard probe chemistries. As there is no thermal cycling, there is no time-constraint on the amplification as there is with PCR and amplification occurs continually. This makes the RPA method significantly faster, with amplification occurring within 3–5 minutes for high copy number samples [15] [16] [27].

The low RPA incubation temperature and high speed makes assay systems based on the RPA technology particularly amenable to field-use due to the low power requirements. This allows detection on simple portable devices, which can be small and lightweight [15] [16], including both miniaturised isothermal real-time detectors and fully automated rapid point-of care devices [15] [16]. RPA and other isothermal methods have shown high tolerance to inhibitors present in crude preparations of patient samples and arthropod vectors [28] [29] [30]. The elimination of laboratory intensive extraction procedures simplifies and speeds up the assay set-up and streamlines the automated point-of-care device, potentially making it lighter and cheaper to both purchase and run.

The aim of this work was to develop an RPA assay as an alternative to the existing RT-PCR methods [31] and provide a fast and fieldable diagnostic, which could be used to test for CCHFV with minimal sample preparation, allowing surveillance and public health management decisions in isolated regions. The RPA shows a high degree of flexibility, so it could also be used in a clinic setting or a traditional laboratory, providing very rapid turnaround of results (<20 minutes), with the potential for high-throughput in a 96-well plate format. The aims of the study were successfully achieved, with the development of a rapid RPA-based isothermal diagnostic capable of detecting all 7 S-segment phylogenetic clades of CCHF within 10 minutes.

## Materials and methods

### Ethics statement

Sera samples tested in this study were previously taken for the laboratory diagnosis of CCHFV and were classified as clinical specimens. No samples were collected specifically for this work, thus ethical approval for the study design was not required. Samples were anonymised within Tajikistan, so investigators were only supplied with sequentially numbered samples. Sera samples were collected and stored within the Tajik Research Institute of Preventive Medicine, Tajikistan. Samples for testing at Public Health England, UK were sent in accordance with national guidelines for both Tajikistan and the UK.

### Primer and probe design

The CCHF RPA assay was designed using alignments of the S-segments of CCHF strains and examination for regions of genomic stability, together with an NCBI BLAST search to check for CCHF specificity. The aim was to find a region which would allow uniform detection of all of the 7 clades of CCHF, but would not detect any non-CCHF sequences. A series of primers and probes were designed within this region and a small selection of synthetic RNA fragments (approximately 1.7kb) were designed to enable primer-probe testing. The primer and probe sets were sequentially tested for detection of the synthetic templates. The lead primer and probe combination was taken forward for further validation.

### Primer and probe preparation

Primers were prepared by Integrated DNA Technologies (IDT) and an RPA EXO probe was synthesised by ATD BIO; all as HPLC purified material. Primer and probe stocks were prepared at 100μM in a Tris-EDTA buffer and diluted to 10μM in molecular grade dH_2_0. A primer mix was prepared to 5μM (of both forward and reverse primers) and both primer mix and probe stocks were frozen at -20°C in single use aliquots.

### Synthetic CCHF S-segment RNA template preparation

Synthetic CCHF S-segment DNA fragments from a selection of Europe group 1 and 2 strains and Africa 1 and 3 strains were prepared by IDT (AY277672, position 1–1673, DQ211638, position 1–1659, DQ211643, position 1–1671, NC005302, position 1–1672 and U88411, position 1–1686) with the addition of T7 and SP6 promoters at the 5’ and 3’ ends respectively (see S1 Fig). RNA templates were prepared from the synthetic DNA using a T7 High Yield RNA synthesis kit (NEB). Approximately 1μg of DNA was added per reaction to a 0.2ml PCR tube with 2μl each of 100mM ATP, GTP, UTP and CTP, 2μl RNA polymerase mix, 2μl 10X reaction buffer and sufficient molecular grade dH_2_0 to make the reaction up to 20μl. The reaction was incubated at 37 °C for two hours in a thermocycler. The RNA template was then DNase-treated to remove the original template contamination; 70μl nuclease free dH_2_O was added per tube with 10μl 10X DNase I buffer and 2μl RNase-free DNase I (NEB). The tubes were mixed and incubated for 15 mins at 37°C. The RNA was purified using a Qiagen RNeasy minikit and quantified using a Qubit broad range RNA kit (Thermo-Fisher Scientific).

### Crude sample preparation

Crude samples included human serum male AB (Sigma H422-20ML), female *Ixodes ricinus* ticks (Charles River) and Surine standard -ve control urine (Sigma S-020-50ML). Sample preparation included aliquoting of the human male serum and urine standard into single use aliquots and storage at -20°C and fridge temperature respectively. The tick samples were prepared as tick pools (10 ticks) and frozen at -20 °C. The ticks were prepared by adding 300μl molecular grade water, transferring to Precellys-R tubes and homogenising using a Precellys tissue homogeniser (3X 20 seconds, with 30 second breaks). The homogenate was centrifuged for 5 minutes at 5900*xg* and the supernatant retained. A serial dilution of each of the neat samples was prepared by diluting in molecular grade water.

### CCHF RPA on the Applied Biosystems 7500 platform

The CCHF RPA assay was performed in a 50μl volume using a TwistAmp Exo-RT kit (TwistDx Cambridge UK). A master mix was prepared, composed of the following/reaction; 4.2μl of a 5μM primer mix (forward and reverse primer), 0.6μl of the 10μM Exo-probe, 29.5μl rehydration buffer and sufficient distilled water to make the reaction up to 50μl after addition of all assay components. Where crude samples were used, 20 units (0.5μl) of an RNase inhibitor was also included (RNaseOUT 40U/μl Invitrogen). The master mix was distributed into the wells of a 96-well PCR plate. 1–5μl of template (together with 5μl crude sample if used) was added and the reaction mixture combined with the lyophilised enzyme pellet, before returning to the plate. The supplied magnesium acetate was diluted to 140mM with molecular grade dH_2_0, 5μl was added to each well and the plate briefly centrifuged before running at 40 °C for 40 minutes on an Applied Biosystems 7500 real-time PCR system, with fluorescence detection every 60 seconds in the FAM channel. The threshold was set at 50,000 delta Rn.

### CCHF RPA with an RT-RPA basic kit

The RT-RPA basic assay was performed in a 50μl volume using a TwistAmp Basic-RT kit (TwistDx Cambridge UK). A master mix was prepared, composed of the following/reaction; 4.2μl of 5μM primer mix (forward and reverse primer), 29.5μl rehydration buffer and sufficient distilled water to make the reaction up to 50μl after addition of all assay components. The master mix was distributed into 0.2ml PCR tube strips. 5μl of template was added and the reaction mixture combined with the lyophilised enzyme pellet, before returning to the wells. The supplied magnesium acetate was diluted to 140mM with molecular grade dH_2_0 and 5μl was added to each well. The tube strip was then briefly centrifuged before incubating at 40 °C for 40 minutes on a thermocycler. The products of the RPA were purified using a Qiagen QIAquick PCR purification kit, then run on an Invitrogen 2% Agarose gel (E-Gel EX with Sybr Gold II) with an Invitrogen E gel 1Kb plus ladder.

### CCHF RT-PCR on the Applied Biosystems 7500 platform

This method was adapted from the paper by Atkinson et al 2012 [31]. Briefly, the CCHF RT-PCR assay was performed in a 20μl volume using a Superscript III Platinum One-step quantitative RT-PCR kit (Invitrogen). For each reaction, a 15μl master mix was prepared containing 10μl of the supplied 2X reaction mix, 0.8μl of superscript III RT/Taq enzyme mix and final concentrations of each primer of 1.2μM and probe of 0.8μM. Where crude samples were used, 20 units (0.5μl) of an RNase inhibitor was also included (RNaseOUT 40U/μl Invitrogen) and sufficient molecular grade water was added for a final reaction volume of 20μl, once all assay components were included. The master mix was distributed into the wells of a 96-well PCR plate. 3–5μl of template (together with 5μl crude sample if used) was added and the plate briefly centrifuged before being run on an Applied Biosystems 7500 real-time PCR system. The PCR run parameters included a 10 minute RT step at 50 °C, followed by a 2 minute denaturation step at 95°C, an amplification stage composed of 45 cycles of denaturation: 95°C for 10 seconds, and annealing/extension at 60°C for 40 seconds, followed by a final extension at 40 °C for 20 seconds. Fluorescence was detected in the FAM channel, once each cycle during the amplification stage. The threshold was set at 250,000 delta Rn.

### CCHF positive viral panel

A collection of CCHFV strains representing each of the following S-segment clades: Asia 1, Asia 2, Africa 2, Africa 3 and Europe 1 were cultured and viral RNA was extracted using a standard RNA extraction kit (QIAamp viral RNA kit). Synthetic whole S-segment viral RNA was used to represent Africa 1 and Europe 2 clades.

### Negative viral panel

Viral RNA extracts covering *Mammarenavirus*, *Marburgvirus*, *Henipavirus*, *Flavivirus*, *Alphavirus* and the *Orthohantavirus genera* were donated by the Rare and Imported Pathogens Laboratory, PHE Porton from a collection of standard diagnostic assay positive controls. They are described as positive, with a Ct of approximately 30 in their respective real-time assays, or as having a clear and distinct band after electrophoresis of PCR products following a conventional block-based PCR. The *Orthonairovirus* samples Hazara and Issyk-Kul were supplied in-house by the National Collection of Pathogenic Viruses and the Virology and Pathogenesis group respectively and were confirmed positive using a block-based PCR.

### Preparation of RNA extracts from field samples for testing on the RPA

Ticks identified as *Hyalomma anatolicum* were collected from the Gisar, Hissor, Kulob, Rumi, Jillikul and Hamadoni districts of Tajikistan and prepared as pools of 10 ticks by the Tajik Research Institute of Preventive Medicine, Dushanbe, Tajikistan. The ticks were added to 1ml Qiagen AVL buffer and were homogenised using a disposable plastic pestle and mortar, allowing 10 minutes post-homogenisation for inactivation. Sera samples were collected from suspected CCHF patients from the Rudaki, Nahiyeh Voseh, Bokhtar, Kologh, Nahiyey farkhor, Moominabad, Khatlon, Bolgevon, Tursonzadeh, Kabadian, Dangarah, Vosse and Kulob districts of Tajikistan by the Tajik Research Institute of Preventive Medicine, Dushanbe, Tajikistan. The sera samples were heat-inactivated for 30 minutes at 56°C and both the inactivated tick pool samples and sera were frozen at -80 for storage. The tick homogenate was centrifuged at 4000*xg* for 10 minutes to pellet the particulate matter. A 140μl volume of the neat sera samples and tick pool supernatant were extracted using a Qiagen QIAamp viral RNA mini kit, with elution into 80μl elution buffer. Note that most sera samples represent a single sera collection, taken on a single day from separate patients. Where samples are multiple sample collections, taken on separate days from a single patient, the sample number is followed by a letter (e.g. sample 4a, 4b, 4c) and a collection number is given.

### Accession Numbers

Genbank accession numbers/ NCBI reference sequences for genes used in this study: Bagdad 12 (Genbank accession number AJ538196), Dubai 616 (Genbank accession number JN108025), DAK 8194 (Genbank accession number U88411), Semunya (Genbank accession number DQ076413), Congo 3010 (Genbank accession number DQ144418), SPU4/81(Genbank accession number DQ076416), IbAr10200 (NCBI reference sequence NC_005302), Kosovo Hoti (Genbank accession number DQ133507), ROS/T128044 (Genbank accession number AY277672), Drosdov (Genbank accession number DQ211643), AP92 (Genbank accession number DQ211638).

## Results

### Assay design

**Fig 1:**
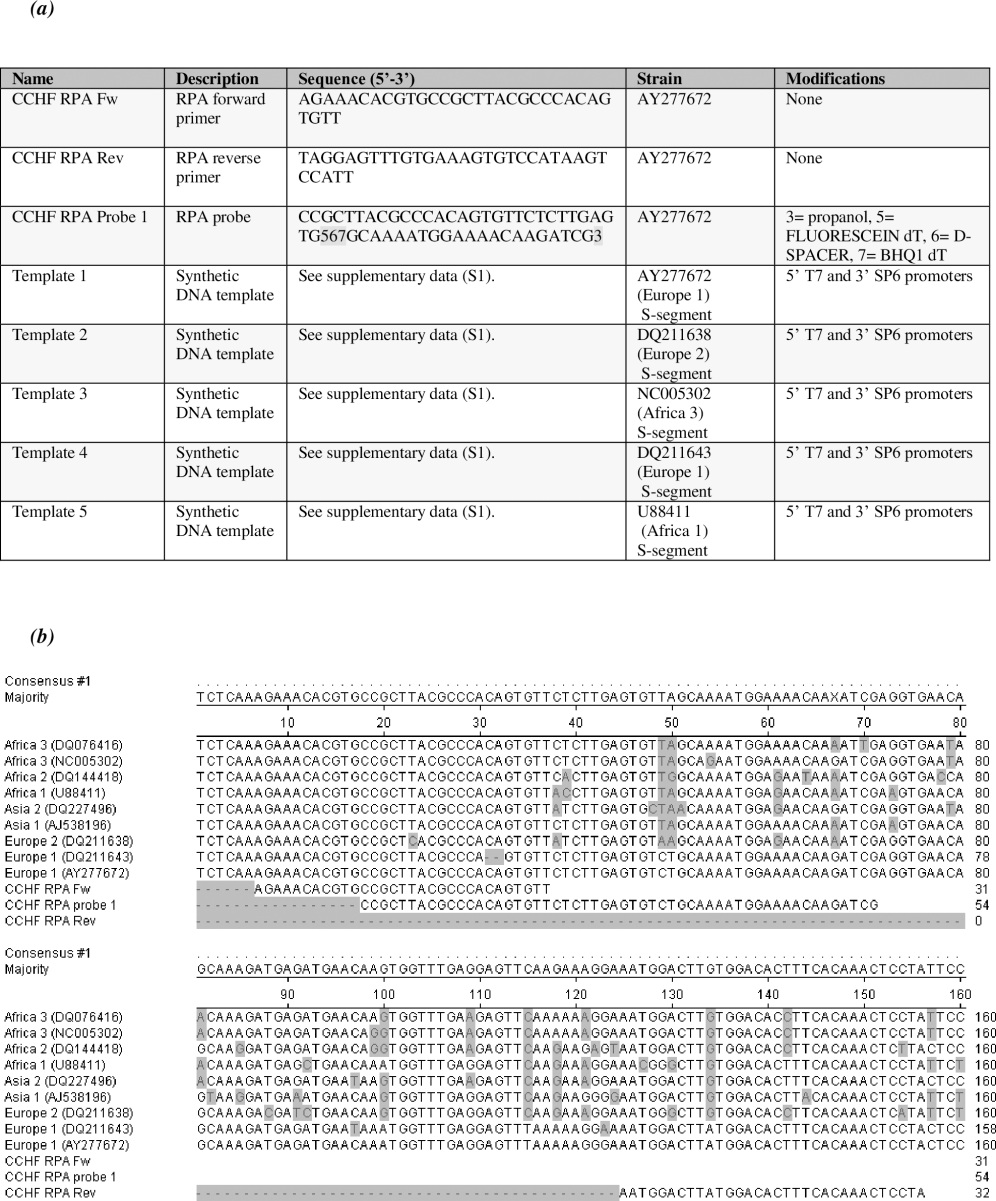
Assay design. **(a) Primer, probe and synthetic template DNA sequences.** Table showing primer and probe sequences and information on the synthetic DNA templates which were used as transcription templates to create the RNA assay controls. Please see supplementary data for the full DNA sequences of the synthetic DNA templates. **(b) Alignment of CCHF strains at the assay design region.** Alignment of a selection of strains of CCHFV representing all 7 S-segment clades of CCHFV (Africa 1, Africa 2, Africa 3, Asia 1, Asia 2, Europe 1 and Europe 2), with inclusion of the RPA forward primer (CCHF RPA Fw), the reverse primer (CCHF RPA Rev) and the probe (CCHF RPA probe 1).

The CCHFV RPA assay was developed by making alignments of CCHF S-segment sequences and analysing them for stable regions to identify sequences that could be used as the basis for primer and probe design. The lead primer-probe set (see **Fig 1A**) is shown aligned to a selection of CCHFV strains representing the 7 S-segment clades of the virus (Africa 1, Africa 2, Africa 3, Asia 1, Asia 2, Europe 1 and Europe 2 (**Fig 1B**). The regions chosen for the RPA primer design were in the vicinity of the primer binding sites of our in-house CCHF PCR, which has shown good cross-clade detection [31]. Some single base mismatches within the primer and probe regions remained, but this was unavoidable due to the high intrinsic sequence variability of the CCHFV S-segment. We were confident that our RPA assay would show good amplification across the clades however, as there was significant overlap in the design region with the PCR and the RPA primers were generally well matched and long, thereby increasing resilience to minimal base-mismatches. The target region was also subjected to a BLAST (blast.ncbi.nlm.nih.gov) search to check for cross-reactivity with non-CCHFV sequences; no non-CCHFV sequences were identified, suggesting that the assay would be CCHFV-specific.

### Detection limit

**Fig 2:**
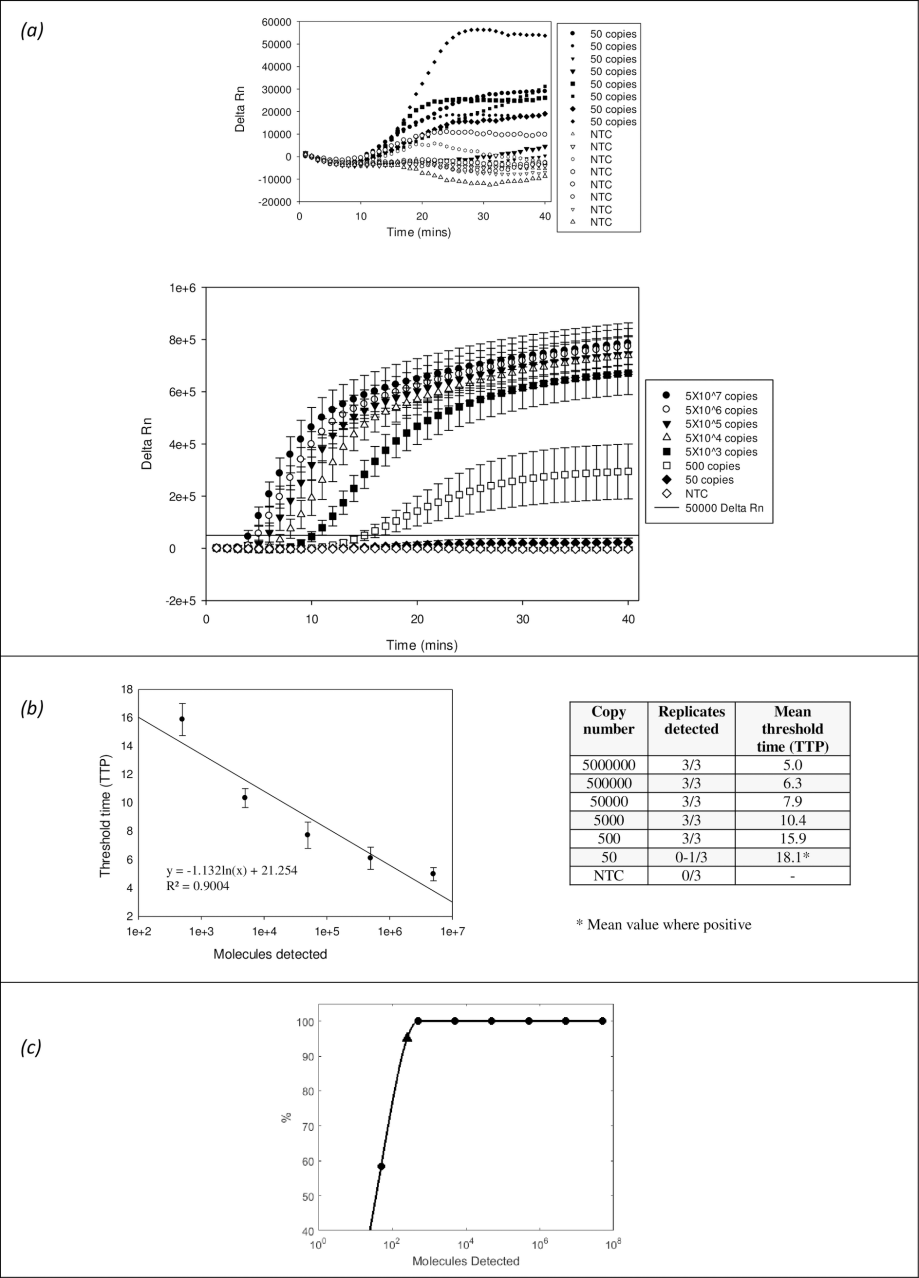
Limit of detection. Limit of detection of the CCHF RPA assay with a serial (1 in 10) dilution of synthetic RNA template of Europe I strain AY277672. The data is represented graphically as **(a)** Delta Rn against time (minutes), in the form of a table of time to positive (TTP value) vs target copy number and as TTP vs target copy number, with a regression line indicating data correlation **(b)**. The data is also shown as a probit analysis performed using the statistical software Matlab for more accurate detection limit approximation **(c)**, with application of a curve-fitting programme (a shape-preserving piecewise cubic interpolation). Note that the probit analysis uses a lower threshold (Delta Rn 18000) to enable the calculation. The values shown for all LOD data are the mean of 8 independent experiments, each of which was performed with three replicates.

The detection limit of the CCHFV RPA assay was determined by testing with a serial dilution of a synthetic RNA S-Segment of the Europe 1 strain AY277672 from 5X10^6^ template copies down to 50 copies (**Fig 2**). Rapid detection was observed, occurring within 5 minutes for the detection of 50X10^6^ template copies, just over 10 minutes for the detection of 5X10^3^ copies and 16 minutes for the detection of 500 copies. The detection limit was between 500 and 50 copies, with strong and rapid detection down to 500 copies, but with variable and sub-threshold detection at 50 copies. The probit analysis gave a predictive value for the limit of detection of this assay as 251 copies of target. The data showed a good correlation between copy number and time to positive, suggesting that this method is either quantitative or semi-quantitative.

### Cross-clade detection of CCHFV

**Fig 3:**
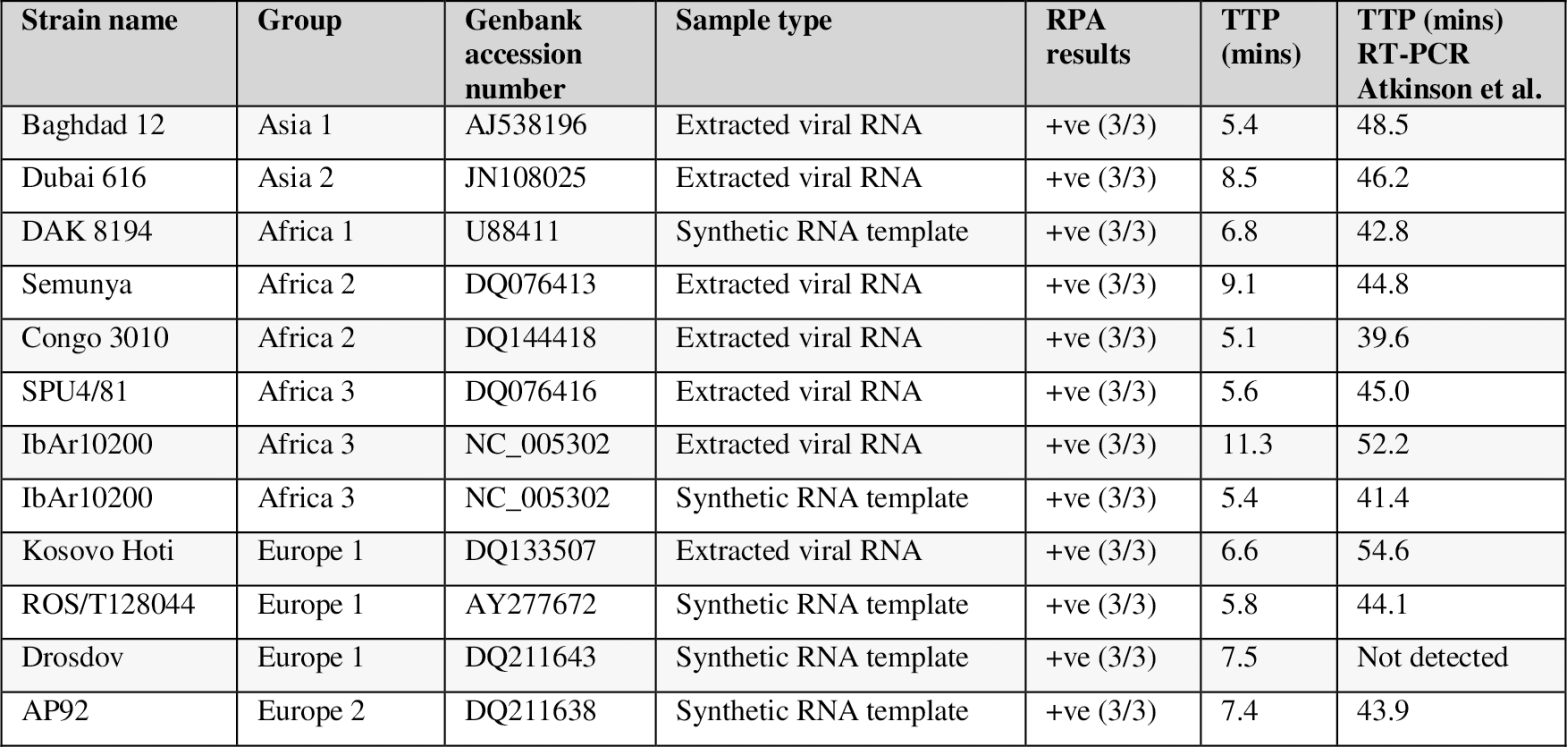
Cross-clade detection of CCHF. Table showing detection of CCHF viral extracts and synthetic CCHF RNA templates by CCHF RPA assay, in the form of TTP (minutes) and number of replicates detected. The synthetic RNA templates were used at 5X10^5^ copies/reaction and viral extracts were confirmed positive by CCHF RT-PCR. The RT-PCR TTP (minutes) are included in the table. Note that the RPA values shown are the mean of 3 independent experiments, performed with 2–3 replicates.

The CCHFV RPA was subsequently tested against a selection of strains representing all 7 extant clades of the viral S-segment. This included where possible cultured viral extract, and a selection of 5X10^5^ copies/reaction synthetic whole S-segment RNA templates (See S1 Fig. for synthetic target sequence information). The target was successfully identified (**Fig 3*)*,** across all 7 clades, with the same rapid detection noted earlier. The RPA time to positive (TTP) was less than 12 minutes for all viral extracts, with most detected between 5–10 minutes. Data from a CCHFV S-segment RT-PCR [31] is shown alongside, with the TTP values ranging from 39.6 to 54.6 minutes. The CCHFV RPA was also performed with a basic RT-RPA kit (no exo-probe) and synthetic CCHFV RNA templates. When analysed by gel electrophoresis with a DNA intercalating dye, the results showed a single band of the expected size of around 150bp, suggesting that only the target region is amplified (see S2 Fig).

### Negative panel testing

**Fig 4:**
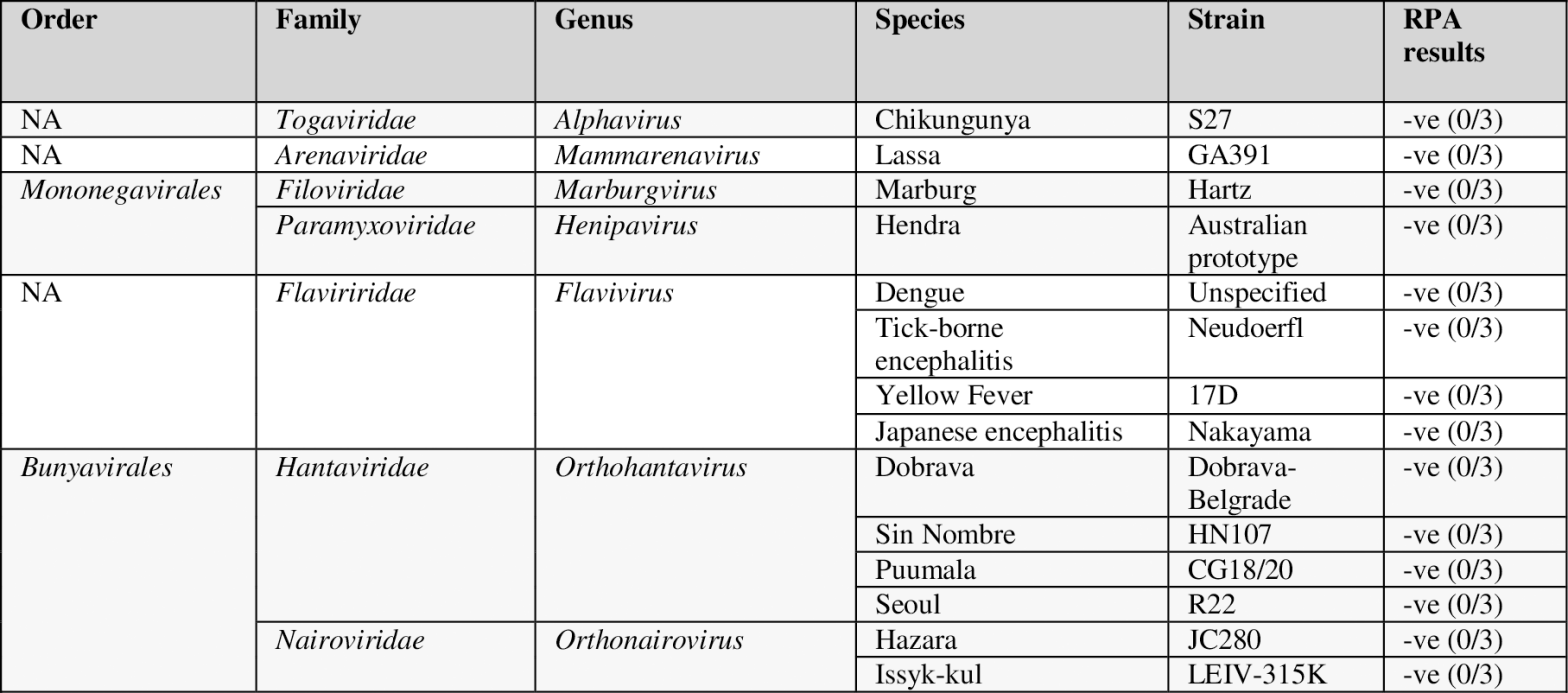
Negative viral panel testing. Table showing the results of a CCHF RPA with a negative viral panel, consisting of a selection of strains from the *Alphavirus*, *Mammarenavirus*, *Marburgvirus*, *Flavivirus*, *Orthohantavirus*, *Henipavirus* and *Orthonairovirus* genera. Results represent 3 separate experiments, each performed with 3 replicates.

The specificity of the CCHFV RPA was tested against a panel of viral extracts, encompassing both phylogenetically related strains and species which cause a similar haemorrhagic aetiology in humans and may be tested as part of a differential diagnosis. These included viruses from the *Mammarenavirus*, *Marburgvirus*, *Henipavirus*, *Orthonairovirus and Orthohantavirus* genera (**Fig 4**). All produced negative results, backing up the hypothesised specificity which had been suggested from the bioinformatic information. Of particular note is the fact that the closely-related *Orthonairoviruses* Hazara and Issyk-Kul were negative.

### Effect of inhibitors in crude sample preparations

**Fig 5:**
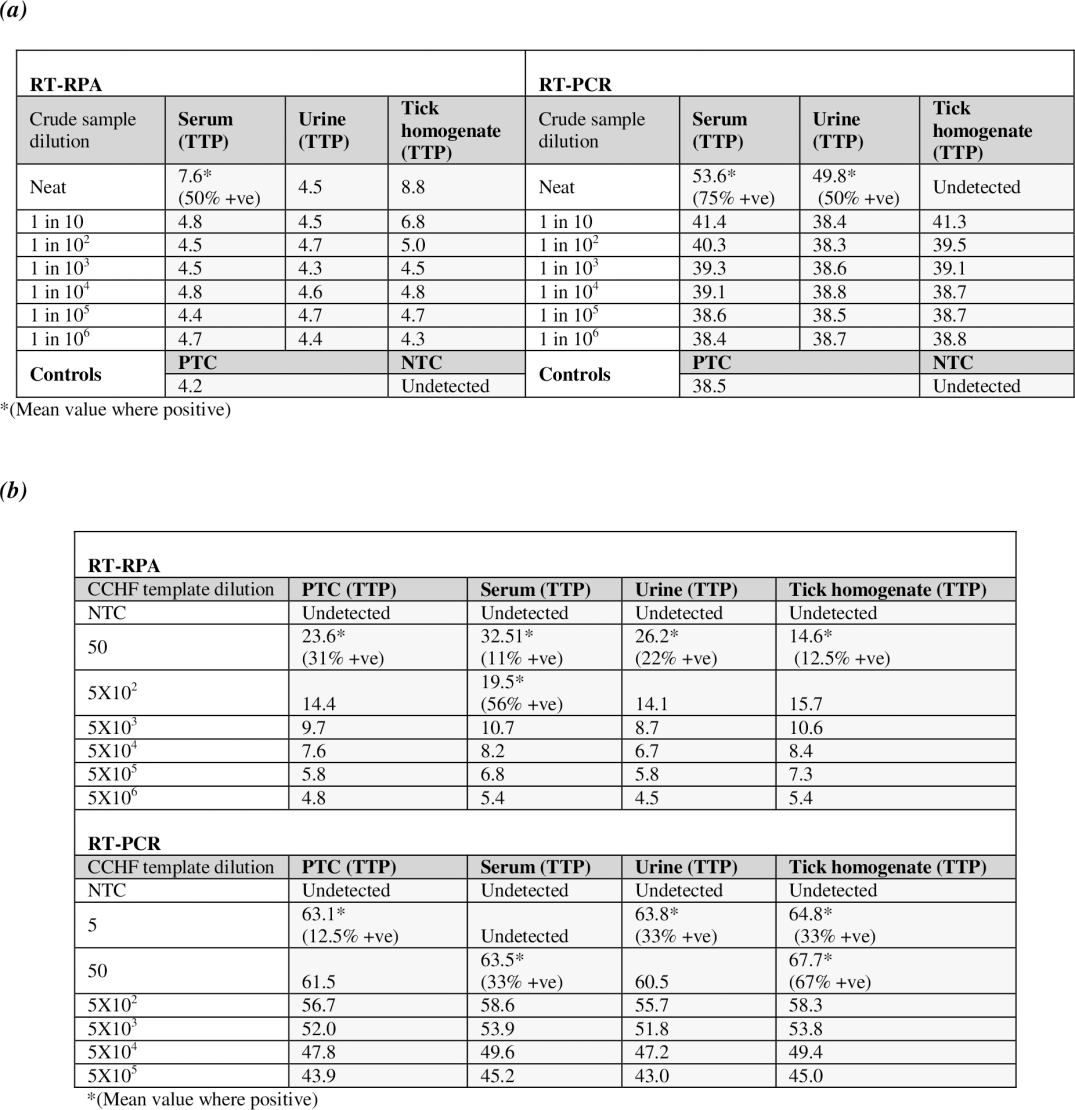
Effect of inhibitors in crude sample preparations. **(a) Effect of crude sample dilution on detection in the RPA assay.** RPA performed with 5X10^6^ copies/ reaction of synthetic RNA template of Europe I strain AY277672 and a serial (1 in 10) dilution of crude preparations of human male serum, urine and tick pool homogenate. **(b) Effect of crude material on assay sensitivity–limit of detection.** RPA performed with a serial (1 in 10) dilution of synthetic RNA template of Europe I strain AY277672 and 1 in 10 diluted crude preparations of serum and urine, or 1 in 100 diluted crude preparations of tick pool homogenate. The RPA results are shown as TTP (minutes) and represent the mean of 3–4 independent experiments, each of which was performed with three replicates. RT-PCR data is also included for comparison, with the values shown representing the mean TTP (mins) of 2–3 separate experiments, each of which was performed with 2 replicates.

Isothermal molecular amplification methods such as RPA and LAMP are tolerant to crude samples, allowing detection in patient bodily fluids, arthropod preparations and other crude material with minimal processing and avoiding the need for extraction [28] [29] [32] [33] [34]. The inhibitory effect of interfering agents within a crude sample on the CCHFV RPA was studied by spiking a known quantity of synthetic CCHFV template into crude preparations of human serum, a homogenised tick pool and synthetic human urine. **Fig 5A** shows strong detection in the presence of each crude sample. There is however evidence of some inhibition at the higher crude sample dilutions, with higher TTP values, although the urine sample showed no observable inhibition at any concentration. Serum showed full detection of the 5X10^6^ copies/reaction template at the 1-in-10 dilution and the tick preparation at the 1-in-100 dilution. This suggests the CCHFV RPA can tolerate the inhibitors present in crude samples with minimal sample preparation, when the copy number of target is high.

We next examined the limit of detection in minimally diluted crude samples. **Fig 5B** indicates that the urine and tick preparation had no effect on the assay limit of detection, with both showing full detection down to 500 copies and partial detection at 50 copies, with comparable TTP values to data using un-spiked purified RNA. Serum showed a minimal effect on the detection limit, with partial detection at 50 and 500 copies and higher TTP values, but with comparable TTP and 100% detection at 5000 copies. Overall however this work suggests that the inhibitory effect of diluted crude samples on the CCHFV RPA is minimal. All crude sample preparations except for the urine showed some inhibitory effect on the detection limit of the CCHFV RT-PCR, with approximately a 10-fold change for the tick and serum preparations.

### Testing of 2013–2015 CCHF outbreak samples

**Fig 6:**
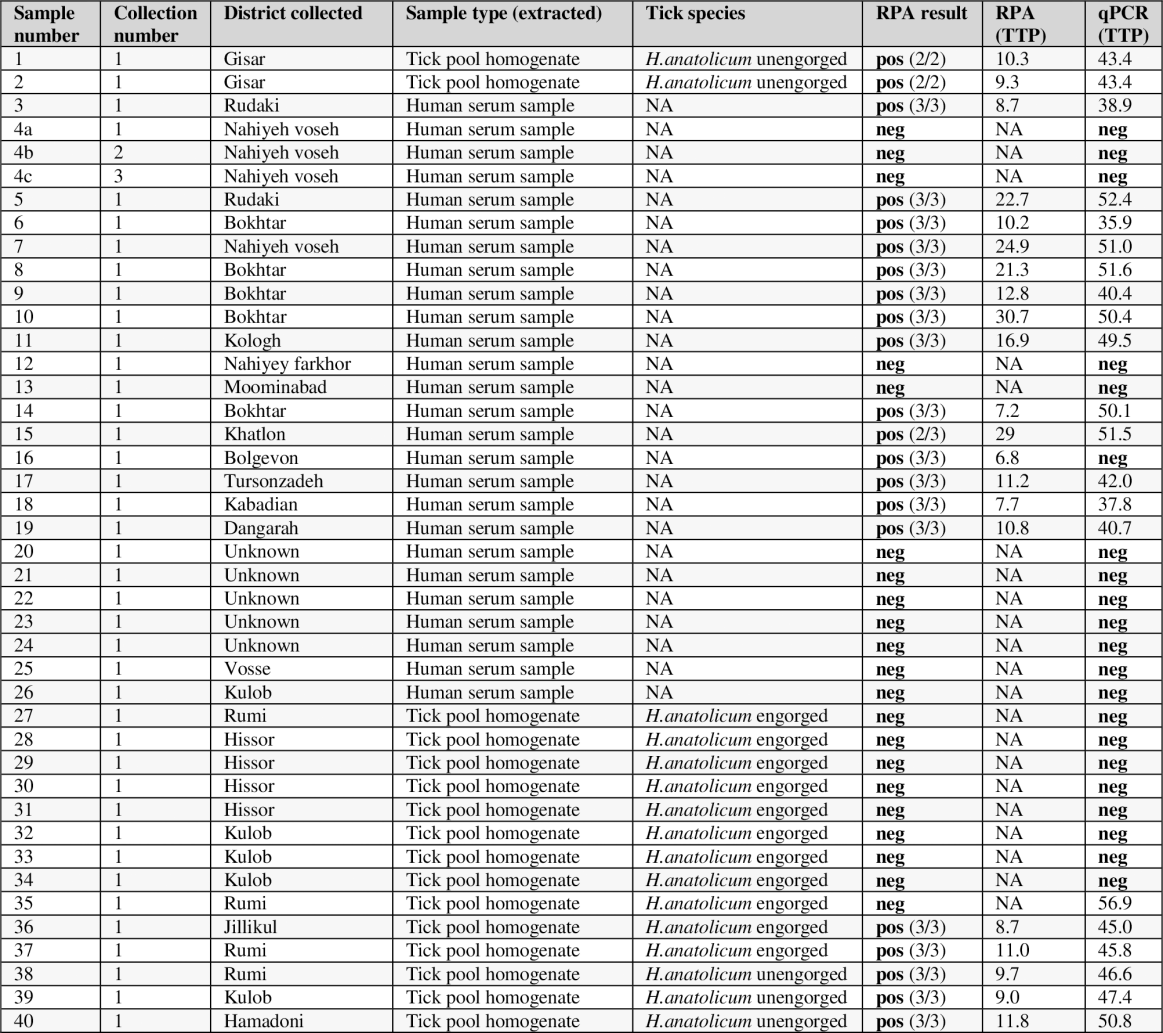
Testing of field samples from Tajikistan CCHF outbreaks between 2013 and 2015. CCHF RPA testing of a collection of field samples from the 2013–2015 CCHF outbreaks in Tajikistan. Sample types include extracted human sera and extracted tick pool homogenate. The RPA results are shown as TTP (minutes) and number of replicates detected. RT-PCR results are shown as TTP (minutes).

CCHFV has caused seasonal outbreaks [35] of disease in Tajikistan since its discovery in the region over 40 years ago. The assay including a portable incubator and reader (Optigene Genie III) was taken to a collaborating laboratory in Dushanbe; the Tajik Institute for Preventive Medicine. This Ministry of Health institute undertakes diagnostic testing for a range of serious pathogens and is the National Centre for CCHF diagnosis; it has regular access to CCHF clinical samples and supports national disease reporting programmes. The CCHFV RPA was set up and used on a collection of extracted patient sera samples and environmental tick extracts obtained in relation to outbreaks of CCHF in 2013–2015 in Tajikistan, these field samples were tested alongside a standard RT-PCR assay [31] that had already been established. The field samples were then sent back to our UK-based laboratory for further testing. The CCHFV RPA (**Fig 6**) detected nearly all confirmed positives (88% of 8 positive tick samples and 100% of 13 positive sera samples), missing only one very late TTP weakly positive tick extract. The RPA also verified the negatives (100% of 8 negative tick samples and 91% of 11 independent (from separate patients) negative sera samples), with one exception (sample 16). It is possible this could be a false positive, however this was a sample which had previously been tested by ELISA and found to be IgM positive for CCHF, so it is likely that this is actually a positive sample. The majority of positive samples were detected in less than 20 minutes (4 samples were detected between 21 and 31 minutes).

## Discussion

A CCHFV RPA has been successfully developed and shown to rapidly detect all 7 CCHFV S-segment types, despite the presence of a small number of single mismatches within the primer and probe binding sites.

Data from a negative panel of closely related viruses including those that might be included as part of a differential diagnosis, illustrate the high specificity for CCHFV. The sensitivity of this assay was statistically estimated to be 251copies of RNA target, which compares well with other published RPA assays [20] [21] [23] [24]. While this sensitivity is lower than the published RT-PCR for CCHFV of 5–50 copies [31], it is still within the clinical detection range present in most acute CCHF patients, as demonstrated by its ability to pick-up all of the confirmed sera positives in the panel of samples from an outbreak in Tajikistan. The assay is also very rapid, providing clear results within 20 minutes (5 minutes for the detection of 50X10^6^ template copies, 10 minutes for the detection of 5X10^3^ copies and 16 minutes for the detection of 500 copies) and an average of 13.8 mins (range 6.8–30.7 mins) for the real clinical/field samples. The CCHFV RPA performed well with spiked crude samples with only a small level of dilution required to remove inhibitory effects. In addition the CCHF RPA strongly detected a clinical sample which had been missed by the PCR, suggesting that the RPA may be able to detect a wider cross-section of CCHF strains than the existing PCR. This is supported by our cross-template detection study, in which the RPA detected a strain Drosdov, which the PCR failed to pick up.

The CCHF RPA has demonstrated its potential for use both as a field/low resource laboratory diagnostic and traditional laboratory test. It is significantly faster than existing RT-PCR-based methods (maximum run time of 35 minutes, compared to 1 hour 10 minutes of the RT-PCR) enabling faster-turnaround and key information to physicians and health care workers, underlining its suitability as a point-of-need diagnostic in the community clinic, or the field. The fast turnaround would also be of benefit in a standard laboratory set-up enabling high-throughput testing. The potential of this diagnostic in low resource settings is highlighted by its validation in Tajikistan using a simple portable isothermal real-time detector. This mirrors published literature generated with the Genie III device [36] and underlines the benefits of an isothermal diagnostic over the traditional RT-PCR in low resource settings.

The applicability of the RPA and other isothermal molecular assays to crude unprocessed clinical samples is often highlighted [28] [29] [30] as a useful advantage. While we show that the current assay is amenable to such samples, further studies will be needed to fully evaluate the potential for use of this assay with crude clinical material, including careful consideration of the biosafety implications when handling this HG4 virus. This will involve testing the RPA with live virus preparations spiked into crude sample material in addition to un-extracted virus in real clinical samples, to confirm whether native virus can be detected. The primary consideration when working with infectious viral material will be to examine methods for disrupting the viral envelope to allow access to the viral nucleic acid and to inactivate the virus to render it safe to handle, whilst avoiding adding to the complexity of the process.

## Supporting information

S1 Fig Sequences of synthetic CCHF S-segment DNA fragments with SP6 and T7 promoters. Shown are the DNA fragments (5’-3’ orientation) designed to be a template for in vitro transcription to create the synthetic RNA templates used to test the RPA. The fragments are composed of a section of the S-segment of CCHF (in black), a T7 promoter (in blue) and an SP6 promoter (in green), flanked by GC-rich tails. **(a) AY277672 S-segment DNA fragment, (b) DQ211638 S-segment DNA fragment, (c) DQ211643 S-segment DNA fragment, (d) NC005302 S-segment DNA fragment, (e) U88411 S-segment DNA fragment.**
(TIF)

S2 Fig CCHF RPA basic DNA gel. Gel showing the products of a basic RT-RPA, (following PCR clean-up) performed with synthetic RNA fragments from a selection of CCHF strains; AY277672, DQ211638, NC005302 and DQ211643.
(TIF)
