# Supplementary figures and images for: A recombinase polymerase amplification assay for rapid detection of Crimean-Congo Haemorrhagic fever Virus infection
Source: PLoS Negl Trop Dis. 2017 Oct 13;11(10):e0006013. doi: 10.1371/journal.pntd.0006013 (PMC5656326; doi:10.1371/journal.pntd.0006013)

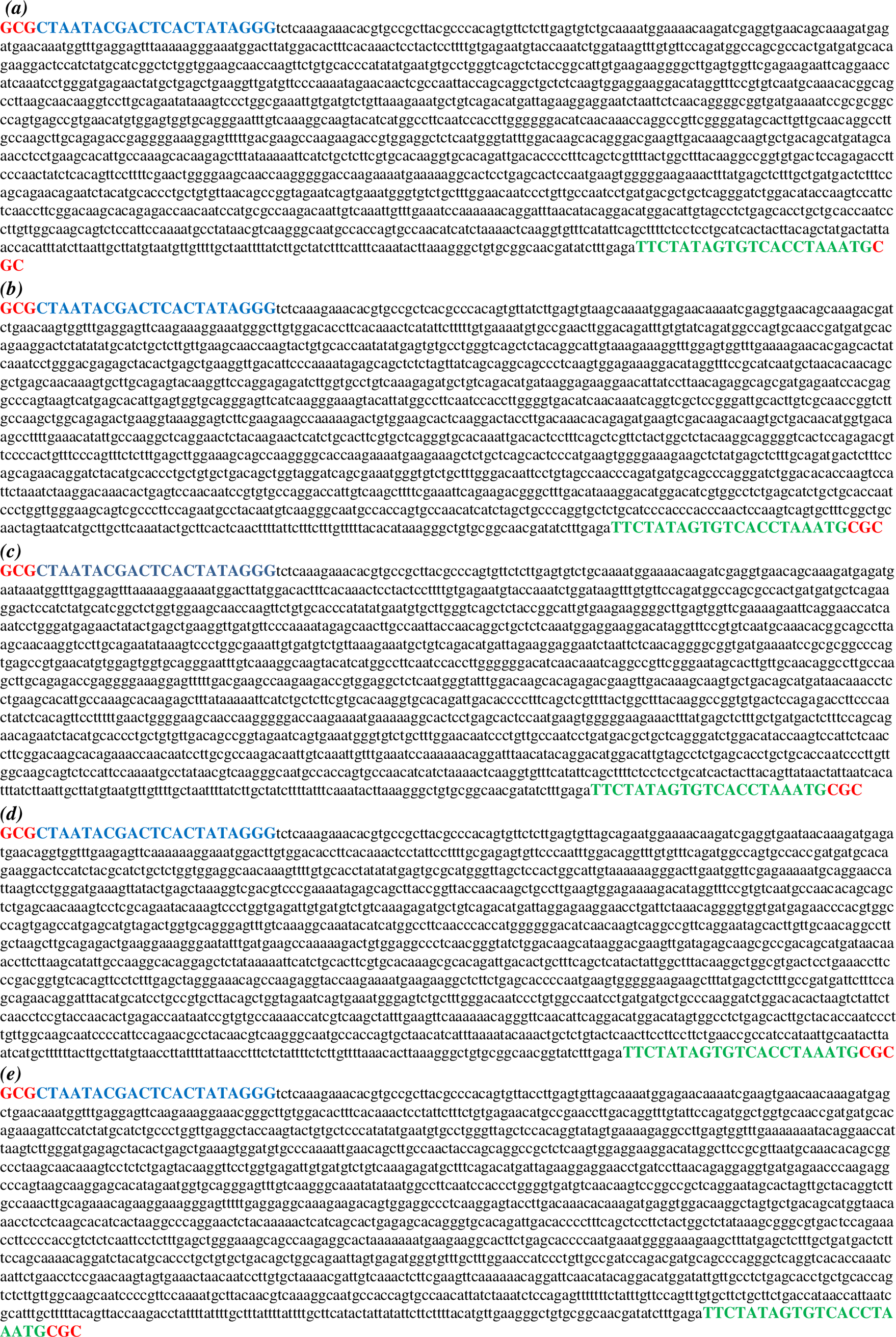

Supplement: S1 Fig — Shown are the DNA fragments (5’-3’ orientation) designed to be a template for in vitro transcription to create the synthetic RNA templates used to test the RPA. The fragments are composed of a section of the S-segment of CCHF (in black), a T7 promoter (in blue) and an SP6 promoter (in green), flanked by GC-rich tails. (a) AY277672 S-segment DNA fragment, (b) DQ211638 S-segment DNA fragment, (c) DQ211643 S-segment DNA fragment, (d) NC005302 S-segment DNA fragment, (e) U88411 S-segment DNA fragment. (TIF) [file pntd.0006013.s001.tif]

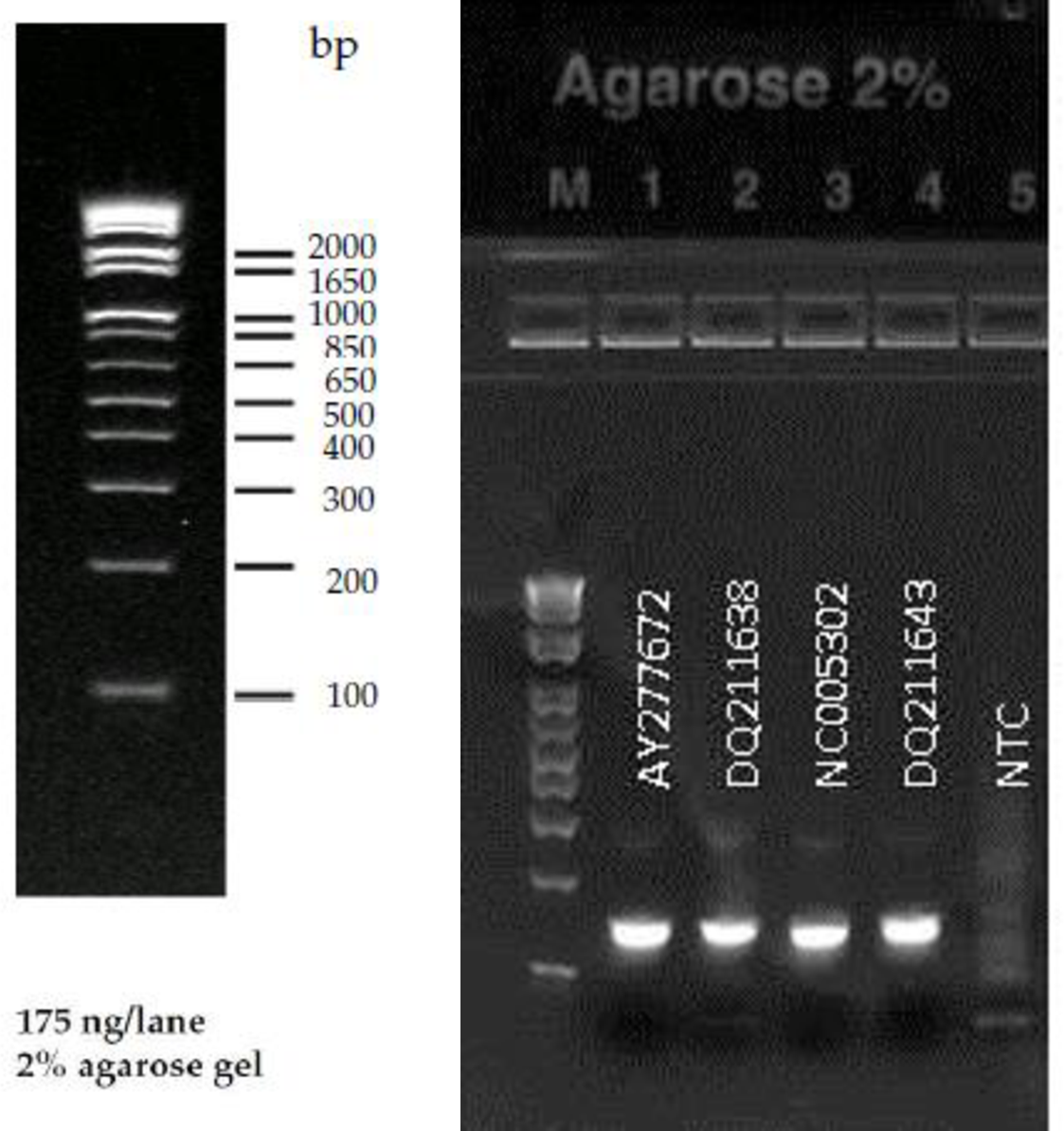

Supplement: S2 Fig — Gel showing the products of a basic RT-RPA, (following PCR clean-up) performed with synthetic RNA fragments from a selection of CCHF strains; AY277672, DQ211638, NC005302 and DQ211643. (TIF) [file pntd.0006013.s002.tif]
